# Amelioration of behavioral and neural deficits in animal models of neurodegenerative disease by nanoformulations of curcumin and quercetin

**DOI:** 10.4103/NRR.NRR-D-25-00343

**Published:** 2025-08-13

**Authors:** Bridget Martinez, Philip V. Peplow

**Affiliations:** 1 Department of Pharmacology, University of Nevada-Reno, Reno, NV, USA; 2 Department of Medicine, University of Nevada-Reno, Reno, NV, USA; 3 Department of Anatomy, University of Otago, Dunedin, New Zealand

**Keywords:** animal models, behavioral deficits, curcumin, inflammation, nanoformulations, neural deficits, neurodegeneration, oxidative stress, quercetin

## Abstract

Neurodegenerative diseases are increasing in prevalence due largely to aging populations worldwide and improved medical care for the elderly. Currently approved drugs can reduce some of the symptoms of neurodegenerative diseases but cannot cure them. Inflammation is involved in the development and progression of neurodegenerative diseases, and oxidative stress is implicated in neurodegeneration associated with cognitive decline and age-related cognitive impairment. Polyphenols such as curcumin, quercetin, and resveratrol possess potent anti-inflammatory and antioxidant properties. Nanoformulations of curcumin and quercetin can optimize their pharmacological effects in the treatment of neurodegenerative diseases. Nanocarriers play a crucial role in delivering drugs across the blood-brain barrier, thereby lowering the risk of peripheral side effects. Various nanoforms have been developed to induce bioavailability and solubility of curcumin and quercetin, including nanoparticles and nanoemulsions. The studies reviewed included 17 using curcumin nanoformulations and seven with quercetin nanoformulations and were tested in widely used animal models of Alzheimer’s disease, Parkinson’s disease, Huntington’s disease, and multiple sclerosis. Many of the curcumin and quercetin nanoformulations brought about improvements in learning and memory in behavioral tests of Alzheimer’s disease models and were effective in reducing oxidative stress in the brain. Both nanocurcumin and nanoquercetin decreased the levels of inflammatory markers in the brain. Nanocurcumin formulations improved motor behavior, gait, and memory in Parkinson’s disease models and increased dopaminergic neurons in the striatum and substantia nigra. Furthermore, nanocurcumin improved locomotor activity, memory, and learning, and the number of dendrites of medium spiny neurons in Huntington’s disease models. Nanocurcumin formulations decreased oxidative stress and inflammation in a model of demyelination. Several important limitations were identified in the studies reviewed and these need to be considered in future studies. Also, clinical trials could be performed using the currently available nanoforms of curcumin and quercetin.

## Introduction

Neurodegenerative diseases are increasing in prevalence due largely to aging populations worldwide and improved medical care for the elderly. The world’s older population is forecast to rise up to 22% by 2050. Current drug treatments can reduce some of the effects of neurodegenerative diseases but cannot cure them. Their use can also bring about undesirable complications. Inflammation is an important player in the development and progression of neurodegenerative diseases as indirect damage to neurons produces inflammatory mediators, which can contribute to the pathological processes of the diseases (Tuppo and Arias, 2005; Cuello, 2017). Some non-steroidal anti-inflammatory drugs, such as meloxicam, which act by inhibiting cyclooxygensase-2 and lipoxygenase, have been used as neuroprotective agents in different neurodegenerative disease models (Ah et al., 2010). Treatment with non-steroidal anti-inflammatory drugs could inhibit inflammation-driven pathogenesis at early stages of the disease (Harrington et al., 2011). Novel nanotechnological formulations with meloxicam have optimized its pharmacological effect in a neurodegenerative disease model and minimized its toxicity in mice (Ianiski et al., 2016; Villalba et al., 2016). Oxidative stress is involved in neurodegenerative diseases associated with age-related cognitive impairment (Axelsen et al., 2011). Oxidative stress causes alterations in cell structure and function, eventually leading to protein and DNA damage, mitochondrial dysfunction, energy deficiency, tau hyperphosphorylation, and overexpression of amyloid-β (Aβ) peptide (Giraldo et al., 2014). In many neurodegenerative diseases, misfolding and aggregation of proteins are closely associated with the accumulation of toxic proteins (e.g., Aβ and tau protein) that cause cellular dysfunction, loss of synaptic connections, and brain damage (Soto and Pritzkow, 2018).

**Figure 1:**
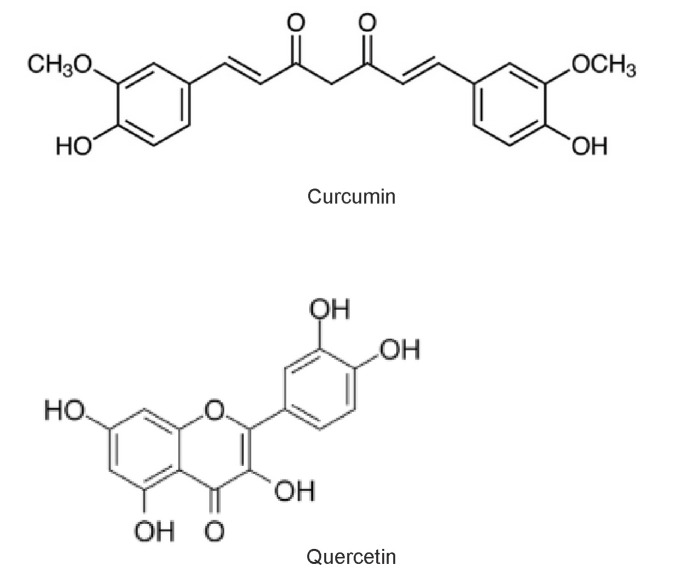
Chemical structure of curcumin and quercetin.

Polyphenols such as curcumin, quercetin, and resveratrol have potent anti-inflammatory and antioxidant properties, as well as antiamyloidogenic properties (Palmal et al., 2014; Bastianetto et al., 2015; Costa et al., 2016; Amanzadeh et al., 2019). The anti-inflammatory action of curcumin and quercetin is related to the inhibition of nuclear factor kappa B signaling (National Centre for Biotechnology Information, 2025) and the reduction of proinflammatory cytokines such as interleukin (IL)-1β, IL-6, and tumor necrosis factor alpha (TNF-α) (Xu et al., 2018). The antioxidant activity of curcumin and quercetin is mediated by the electron-donating groups, especially the phenolic hydroxyl group in their chemical structure (Zheng et al., 2017; **Figure 1**) and by modulating the nuclear factor E2-related factor 2 pathway. These mechanisms and cellular pathways have been recently reviewed (Abdul-Rahman et al., 2024; Alharbi et al., 2025). The mechanism of curcumin acting against neuroinflammation and oxidative stress and its suppression of important genes in Alzheimer’s disease (AD) has been illustrated in a recent report (Abdul-Rahman et al., 2024). Curcumin also has cytoprotective properties (Panda et al., 2017). Similar to meloxicam, nanoformulations of curcumin and quercetin can optimize their pharmacological effects in treating neurodegenerative diseases. Both compounds have low aqueous solubility, high metabolic rate and low bioavailability, rapid systemic clearance, and low ability to cross the blood–brain barrier (Anand et al., 2007; Aggarwal et al., 2009; Nelson et al., 2017). However, these limitations can be overcome by using a nanoformulation of curcumin and quercetin enabling them to cross the blood–brain barrier (Barbara et al., 2017). Interestingly, co-nanoencapsulated meloxicam and curcumin improved cognitive impairment caused by Aβ in mice (Gutierrez et al., 2021).

The development of nanocarriers is important for delivering drugs across the blood–brain barrier (Perche et al., 2017; Mi et al., 2020), reducing the risk of peripheral side effects, thereby providing benefits and greater acceptability to the long-term treatment (Liu et al., 2020b). Only high-fat soluble substances with molecular weights below 500 Da can pass through the blood–brain barrier (Pardridge, 2005a). However, more than 98% of neuropharmaceuticals are unable to meet these requirements (Elias et al., 2001; Pardridge, 2005b; Re et al., 2012; Maussang et al., 2016). Nanocarriers that can cross the blood–brain barrier are exosomes, cerasomes, liposomes, nanoparticles, nanocapsules, and other nanoformulations. Exosomes are cell-derived bilayer lipid vesicles with a diameter of 40–100 nm and are mainly involved in information exchange and substance transfer between cells (EL Andaloussi et al., 2013; Johnsen et al., 2014; Zhang et al., 2015). Having a lipid bimolecular structure similar to the cell membrane provides the possibility for exosomes to efficiently load hydrophobic and hydrophilic drugs (Kim et al., 2018; Qu et al., 2018). Cerasomes have a bilayer vesicular structure and a silicate surface with a molecular structure similar to lipids but possess better morphological stability than conventional liposomes and have better biocompatibility than silica nanoparticles. As a brain-targeting drug-delivery system, liposomes have biocompatibility, stability, enhanced peripheral circulation, and ligands that can be attached to the surface for receptor-mediated drug delivery (Pandian et al., 2022). Lipid-based nanoparticles, because of their lipidic nature, are readily taken up by the brain (Kaur et al., 2008; Aboutaleb et al., 2014) and widely used to increase drug delivery to the brain (Yang et al, 1999). Polymeric nanoparticles for drug delivery have easy synthesis, a large surface area, and stable pharmacokinetics. Their nanosize and ability to be surface modified with various molecules, including ligands, facilitate their crossing of the blood–brain barrier and targeted delivery to the brain. Various methods have been developed to induce the bioavailability and solubility of curcumin and quercetin including nanoparticles, encapsulated liposomes, and encapsulation into poly lactic-co-glycolic acid (Tsai et al., 2011; Amanzadeh Jajin et al., 2021). Brain-targeting drug delivery systems have been recently reviewed (Sun et al., 2025), as well as the assistance of physical techniques such as ultrasound, microwave, and laser (Sun et al, 2021).

Neurodegenerative diseases include AD, Parkinson’s disease (PD), Huntington’s disease (HD), multiple sclerosis, and amyotrophic lateral sclerosis. Characteristic features of AD are the destruction of the functional activity of neurons in different areas of the brain, including those responsible for memory, learning, emotional reactions, and behavior (Nelson et al., 2012). The mechanisms responsible for AD onset and the secondary development thereafter include amyloid plaques and neurofibrillary tangles, which are caused by deposits of Aβ fragments and hyperphosphorylated tau proteins (Qi et al., 2016). The small clusters of Aβ plaque may block the signaling between cells at synapses and then activate the immune cells that cause inflammation and trigger the destruction of neurons (López et al., 2012). PD is a long-term progressive neurodegenerative disease that predominantly influences the motor neurons. PD is distinguished pathologically by an exponential decrease in the number of dopaminergic neurons in the substantia nigra para compacta, as well as the aggregation of ubiquitin and α-synuclein intracellular proteins, in addition to ubiquitin in cytoplasmic bodies called Lewy bodies (Goedert, 2001; Calabrese et al., 2018; Yamasaki et al., 2019). The core pathway affected by the disease is the nigrostriatal pathway (Kordower et al., 2008; Roostalu et al., 2019). The oligomeric and fibrillar misfolded forms of α-synuclein are the neurotoxic species in the pathogenesis of PD. HD is an autosomal dominant progressive neurodegenerative disorder that causes involuntary movements, psychological changes, and cognitive impairment, including dementia (Zuccato et al., 2010). A mutation in the Huntington gene (*HTT*) leads to expansion of the cytosine-adenine-guanine repeats, causing an elongation of the polyglutamine segment in Huntington protein (HTT) and resulting in the mutated form of this protein (mHTT) (MacDonald et al., 1993). The mHTT induces toxicity in neurons and causes profound damage to the medium spiny neurons in the striatum in the early stages of the disease, affecting neurons in the cortex and other brain areas during the later stages (Zuccato et al., 2010). Multiple sclerosis is a chronic inflammatory and neurodegenerative disease characterized by demyelination of nerves and axonal damage (Reich et al., 2018). Impaired cognition, loss of mobility, gastrointestinal and urinary dysfunction, and pain are common complications of multiple sclerosis. Cognitive impairment in multiple sclerosis is considered to result from neuroinflammation and oxidative stress (Moezzi et al., 2022).

Recent studies indicate that the delivery of a nanotized compound is more effective than its parent compound in the brain (Reddy and Labhasetwar, 2009; Vergoni et al., 2009; Hoppe et al., 2013; Tiwari et al., 2013). For example, nanoparticle encapsulation enhances the solubility of curcumin and prolongs its circulation within the body and sustained release in the brain (Tsai et al., 2011). It also helps to modify the metabolism of curcumin (Chopra et al., 2021). The enhanced efficiency of nanoforms with respect to molecular forms is due to increased chemical stability, enhanced water dispersibility, enhanced bioavailability, and multivalent interaction (Pradhan et al., 2018). Nanoparticle forms of green tea polyphenol and curcumin have performances that can be enhanced up to 100,000 times than the respective molecular forms (Pradhan et al., 2018). This review aims to assess nanoforms of curcumin and quercetin in animal models of neurodegenerative disease and which might have translational and clinical value in treating neurodegenerative diseases. While no single animal model can reproduce all the pathological and behavioral features of human patients with neurodegenerative diseases, the established animal models can replicate to some extent some of these important features.

## Methods and Results

### Search strategy for curcumin nanoforms

**Figure 2:**
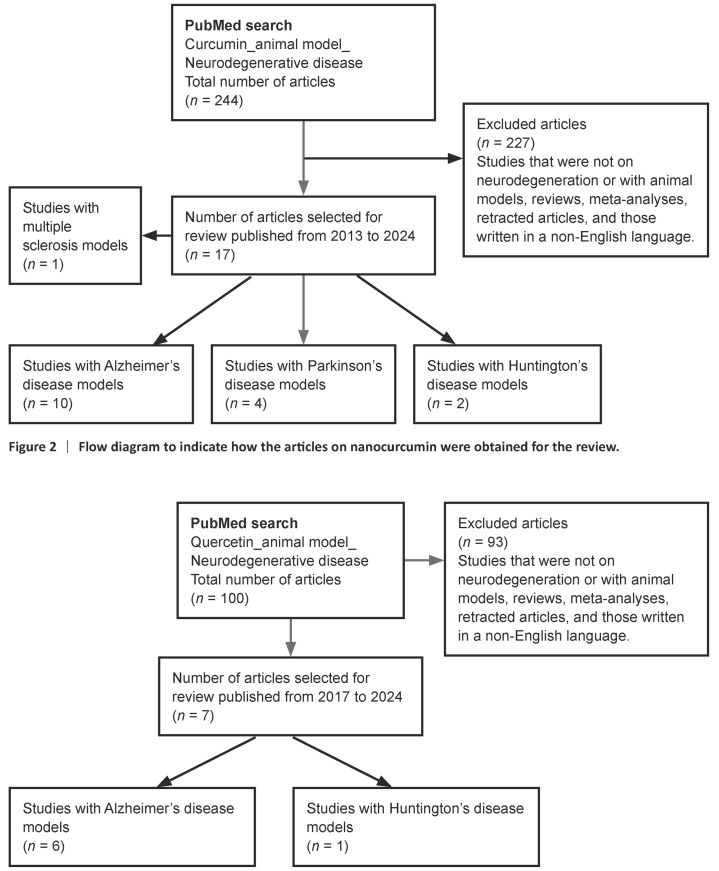
Flow diagram to indicate how the articles on nanocurcumin were obtained for the review.

We performed a PubMed search for curcumin nanoforms for treating neurodegenerative diseases using the search terms “curcumin,” “animal model,” and “neurodegenerative disease.” The total number of articles found was 244 and these articles were published from July 2008 to June 2024. Of these articles, 17 were selected for the review; those not selected were reviews, meta-analyses, retracted articles, not performed with nanoforms in animal models, not on neurodegeneration, or written in a non-English language. Ten of the chosen articles used AD models, four PD models, two HD models, and one multiple sclerosis model (**Figure 2**).

### Search strategy for quercetin nanoforms

**Figure 3:**
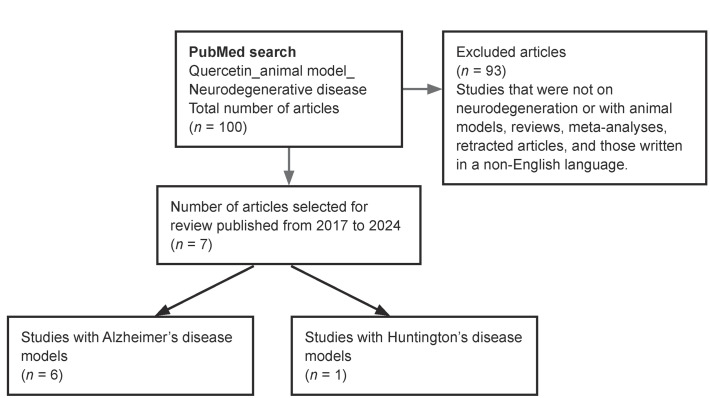
Flow diagram to indicate how the articles on nanoquercetin were obtained for the review.

We performed a similar PubMed search for quercetin nanoforms for treating neurodegenerative diseases using the search terms “quercetin,” “animal model,” and “neurodegenerative disease.” The total number of articles found was 100 and these articles were published from April 2008 to July 2024; those not selected were reviews, not performed with nanoforms in animal models, or written in a non-English language. Of these articles, seven were selected for the review. Six of the chosen articles used AD models, and one was an *in vitro* HD model (**Figure 3**).

The experimental design used in each of the studies reviewed is described below, and the findings are presented in **Tables 1** and **2**.

**Table 1:** Effects of curcumin nanoformulations in animal models of neurodegenerative disease

Curcumin nanoformulation and administration	Animal model of neurodegenerative disease	Behavioral and neurorestorative effects of curcumin nanoformulations in animal models of neurodegenerative disease	Reference
**Alzheimer’s disease**			
CUR-PIP S-SNEDDS.	Swiss albino mice, adult male, 18–22 g. AlCl3 given intragastrically 20 mg/kg/d and D-galactose i.p. 120 mg/kg/d for 40 d.	In novel object recognition test, there were significant improvements in learning and memory in groups treated with CUR-PIP-loaded S-SNEDDS (dose I: 25 mg/kg curcumin + 5 mg/kg piperine; dose II: 50 mg/kg curcumin + 10 mg/kg piperine). In Morris water maze test, a significant decrease in escape latency time was observed in curcumin treatment groups.	Ahmad and Hafeez, 2023
All SNEDDS (self-nanoemulsifying drug delivery system) given by oral gavage for 21 d. SNEDS average droplet size 22–310 nm.			
Curcumin nanoparticles (CN) (purchased from One Planet Nutrition Company, Florida, USA) given for 6 d by oral administration prior to i.c.v. injection of STZ.	Wistar albino rats, adult male, 250–350 g. On the 7^th^ d of administration of CNs, STZ given as a single bilateral i.c.v. injection of STZ (3 mg/kg in artificial CSF) to induce Alzheimer’s disease (Bao et al., 2017).	In Morris water maze test, the latency to locate the platform submerged below the water surface was decreased in animals pretreated with CNs 50 mg/kg/d and nonsignificantly changed from control rats. Daily protection of STZ-induced animal model with CNs for 15 d improved the changes in MDA and GSH levels in cerebral cortex and returned NO levels to nearly control levels. MDA and GSH levels in hippocampus returned to control levels after daily protection with CNs for 15 d, while NO levels showed a small improvement. The increase in TNF-α in the cortex and hippocampus was ameliorated after daily protection with CNs for 15 d. The increased immunoreactivity of tau protein with increased accumulation of neurofibrillary tangles in cortex and hippocampus was attenuated by CNs.	Noor et al., 2022
Lipid core nanocapsules (LCN) loaded with curcumin and meloxicam and treated on d 2, 4, 6, 8, 10, and 12 by intragastric administration. Particle size range was 257–312 nm.	Swiss mice, male, 40–50 g, 3 mon of age, were injected i.c.v. with 3 nmol (3 µL) of aggregated amyloid-β (Aβ) peptide, fragment 25–35.	In the probe test, meloxicam, LCN MEL 5 mg/kg, 17 mL/kg, meloxicam + curcumin association 5 and 10 mg/kg, respectively, 17 mL/kg and LCN MEL-CUR 5 and 10 mg/kg, respectively, 17 mL/kg protected partially against the impairment in memory caused by Aβ peptide infusion; only the meloxicam and curcumin association completely reversed Aβ-induced memory impairment, in both canola oil and nanocapsule formulations. Oil formulations of curcumin as well as meloxicam and curcumin-co-loaded LCN were effective in reducing Aβ-increased COX-2 protein expression levels in the prefrontal cortex.	Gutierrez et al., 2021
Curcumin-primed exosomes (Exo-CUR) given for 7 d by i.p. injection in saline suspension. Exo-CUR had a diameter of approximately 100 nm.	C57BL/6 mice, 5–7 wk, were injected with okadaic acid (OA) 0.2 µM (2 µL) in right dorsal hippocampus.	Exo-CUR-treated mice 100 µg/mL/d curcumin in 0.2 mL showed shorter escape latencies and exhibited spatially oriented swimming behavior; they spent more time in the target quadrant and showed an increased number of crossings, indicating that administration of Exo-CUR improved memory and spatial learning of mice. Expressions of BAX and cleaved-caspase-3 were significantly reduced by the administration of Exo-CUR. When free curcumin was administered there was no notable difference in escape latencies or target quadrant occupancy. As compared with curcumin, Exo-CUR showed better anti-apoptosis effects by inducing lower expressions of BAX and cleaved-caspase-3. The Exo-CUR group had more NeuN-positive cells in the CA1, CA3, and DG region, showing that Exo-CUR treatment could effectively reduce OA-induced neuronal injury.	Wang et al., 2019
Curcumin-loaded nanostructured lipid carriers (CUR-NLCs) given by i.v. injection for 4 d. CUR-NLCs had a mean particle size of 118 nm.	Sprague-Dawley rats, male, 200–220 g, 6–8 wk, were injected with Aβ suspension 100 ng/µL (2 µL) on each side of posterior hippocampus.	In Morris water maze test, escape latency was significantly decreased between 1^st^ and 4th d in both preventive (CUR-NLCs 4 mg/kg/d of curcumin given for 4 d before Aβ injections) and treatment groups (CUR-NLCs 4 mg/kg/d of curcumin given for 4 d after Aβ injections). CUR-NLCs treatment significantly decreased distance travelled to find the escape platform for both the preventive and treatment groups. The time spent in the target quadrant showed a significant increase in both the prevention and treatment groups. The results of the probe test after removing the platform indicated that CUR-NLCs-receiving rats had greater retention of its position. MDA levels were significantly decreased in the treatment group. ROS levels were significantly decreased in CUR-NLCs administration groups. CUR-NLCs administration significantly increased thiol levels with the increase in thiol level in prevention group being significantly higher than the treatment group. The number of Aβ plaques was decreased in the prevention and treatment groups. There was no Aβ deposition in the prevention group.	Sadegh Malvajerd et al., 2019
Curcumin liquid core nanocapsules (LNC) given p.o. for 14 d after receiving Aβ_1-42_. LNC diameter 247 ± 4 nm.	Swiss albino mice, 30–35 g, 18–22 mon, received i.c.v. injection of Aβ_1-42_ 400 pmol/mouse (3 µL).	In Morris water maze test, treatment with free curcumin 50 mg/kg (FC50), curcumin loaded-lipid core nanocapsules 10 mg/kg (LNC10) and curcumin loaded-lipid core nanocapsules 1 mg/kg (LNC1) decreased the cognitive deficit. Treatment with FC50, LNC10 and LNC1 attenuated the increase of mRNA expression of inflammatory markers TNF-α, IL-1β, IL-6, IFN-γ, NF-κB in hippocampus and prefrontal cortex of mice. LNC10 significantly reduced mRNA expression of inflammatory markers IL-1β, IL-6, IFN-γ, NF-κB compared to FC50 in both the hippocampus and prefrontal cortex. Treatment with FC50, LNC10 and LNC1 attenuated the increase in the levels of inflammatory markers TNF-α, IL-1β, IL-6, IFN-γ, NF-κB in hippocampus and prefrontal cortex of mice. LNC10 significantly reduced the levels of inflammatory markers TNF-α, IL-1β, IL-6, IFN-γ, and NF-κB compared to FC50 in both the hippocampus and prefrontal cortex.	Giacomeli et al., 2019
Curcumin liquid core nanocapsules (NLC C) given by intragastric route on d 2, 4, 6, 8, 10, and 12. Particle diameter ranged from 291 to 312 nm.	Swiss mice, male, 40–50 g, 3 mon, received i.c.v. injection of Aβ25-35 3 nmol (3 µL).	A depressant-like behavior, observed by an increased immobility time in the tail suspension test, and forced swimming test, caused by Aβ25-35 was reversed by NLC C treatment 10 mg/mL, 3 µL. Free curcumin 10 mg/mL, 3 µL did not resolve depressant-like behavior induced by Aβ25-35 infusion in both tests. Aβ25-35 administration induced an increase in reactive species levels, SOD and CAT activities in the prefrontal cortex compared to the control group. NLC C treatment reversed all Aβ25-35-induced changes. Free curcumin reversed only CAT activity in the prefrontal cortex. Aβ25-35 administration caused a significant decrease in the SOD/CAT ratio in the hippocampus compared to control group. NLC C caused an increase in hippocampal SOD/CAT ratio.	Fidelis et al., 2019
Novel CUR formulation (NCF) supplied in drinking water for 3 mon.	APPswe/PS1deE9 transgenic mice, 14 mon.	In open field test, NCF-treated (47 mg/kg of curcumin) APPswe/PS1deE9 mice showed significantly longer travel distance, a higher number of rearing, and percent time spent in the central zone compared to without treatment groups. There was no significant difference between the performances of NCF-treated APPswe/PS1deE9 mice compared to WT mice and major improvement was observed compared to basal control group, indicating NCF treatment could reverse the cognitive deficits to the much lower ages for APPswe/PS1deE9 mice. In the Morris water maze test, APPswe/PS1deE9 mice treated with NCF showed a significant reduction in the escape latency time. In probe trial, NCF-treated APPswe/PS1deE9 mice showed a greater number of annulus crossing, and longer time spent in the quadrant having the platform compared to non-treated groups, indicating significant improvement in spatial learning and memory in NCF-treated mice.	Parikh et al., 2018
Curcumin-PLGA-nanoparticles (CUR-PLGA-NPs) given daily for 2 wk by i.p. Particle size 30 ± 5 nm.	Wistar rats, 4 wk, injected with Aβ1–42 2 µg/µL (2 µL saline) into both sides of hippocampus.	Both CUR-PLGA-NPs and bulk curcumin at a dose of 20 mg/kg significantly enhanced hippocampal neurogenesis and reversed Aβ-induced cognitive deficits. Bulk curcumin at a dose of 0.5 mg/kg showed no significant enhancement of neurogenesis, while curcumin-loaded nanoparticles were effective at this dose. There were no significant effects on learning and memory in empty PLGA-NP-treated rats. CUR-PLGA-NPs also inhibited Aβ-induced neurodegeneration in the hippocampus.	Tiwari et al., 2014
Curcumin nanoparticles (NC) administered weekly for 3 mon by gavage. Mean particle size < 80 nm.	Tg2576 transgenic mice, 9 mon.	In radial arm maze test, it was determined whether treatment (before or after treatment) or the treatment condition (NC 23 mg/kg of curcumin, curcumin 23 mg/kg, or control) affected memory. Neither affected contextual memory, but treatment significantly increased cue memory in NC-treated compared to control-treated mice. Compared to control mice, the area occupied in hippocampus by amyloid plaques was significantly less in curcumin-treated mice but not in NC-treated mice.	Cheng et al., 2013
**Parkinson’s disease**			
Curcumin loaded human endometrial stem cells derived exosomes (hEnSCs EXOs-CUR) injected intranasally 10 times every other day for 28 d. hEnSCs EXOs-CUR size ranged 30–200 nm.	C57BL/6 mice, male, 8 wk, 27–32 g, in which 6-OHDA was injected into striatum.	The rotarod test was used to monitor motor behavior of mice. Compared with 6-OHDA group, the rotation number of mice in the free curcumin 10 mg/kg and hEnSCs EXOs-CUR 10 mg/kg group in the 3rd and 4th wk was significantly decreased, although the rotation number in hEnSCs EXOs treated mice showed no rotational behaviors changes. The rotational numbers in the hEnSCs EXOs-CUR group were the lowest among all studied groups. Compared with 6-OHDA group, the residence time of mice treated with free curcumin, hEnSCs EXOs and hEnSCs EXOs-CUR was increased together with elevated motor latency. Mice in the hEnSCs EXOs-CUR group showed the highest residence time on the rotarod among all studied groups. Intranasal administration of hEnSCs EXOs-CUR group showed an approximately 2-fold increase of TH expression neurons in SNpc region compared to the 6-OHDA-PD model mice treated with PBS. Mice treated with hEnSCs EXOs-CUR showed the highest expression of TH gene compared to control group and 6-OHDA-PD model mice. TH expression was higher than free curcumin group and empty hEnSCs EXOs group. Intranasal administration of hEnSCs EXOs-CUR had lower α-synuclein expression level compared to injection of free curcumin. There was a considerable decrease in the expression level of BAX and caspase 3 and a significant increase in BCL2 expression level in mice treated with hEnSCs EXOs-CUR compared to all studied groups. Curcumin loaded EXOs was more effective in reducing apoptosis compared to other experimental groups.	Mobahat et al., 2023
Curcumin analogue nanoformulation (NanoCA) which is self-assembled from CA molecules and polyethylene glycol,	C57BL/6 mice, male, approximately 22 g, injected i.p. with MPTP-HCl (30 mg/kg) daily for 7 d.	In open field test, treatment with NanoCA 5 mg/kg significantly ameliorated the anxiety status and memory dysfunction of mice caused by MPTP. There was an improvement in gait after NanoCA treatment. After NanoCA treatment, the OB neurons were denser in comparison to those of MPTP-treated PD model mice, indicating the protective effect of NanoCA on OB neurons against MPTP toxicity. There was a decrease in TH expression in OB sections of MPTP group but a rescue in the NanoCA group. Histological analysis showed that striatal dopaminergic fibers and TH-positive neurons in substantia nigra of the MPTP group were remarkably reduced and significantly increased after NanoCA treatment. Western blot analysis demonstrated the promoting effect of NanoCA on reducing monomer, oligomer, and aggregates of α-synuclein in the midbrain tissues of PD model mice.	Liu et al., 2020b
given intranasally on d –2, –1, 0, 2, 4, 6, 8, with d 0 being the day of starting MPTP injections. NanoCA size 131 nm.			
Curcumin-loaded polysorbate 80-modified cerasome nanoparticles (CPC NPs) given i.v. Mice treated every 2 d for four times. CPC nanoparticles size 110 nm.	C57BL/6 mice injected i.p. with MPTP (40 mg/kg) daily for 7 d.	A loss of significant motor activities was seen in groups treated with curcumin-loaded cerasomes and blank cerasomes with UTMD. CPC NPs with 5% PS 80 in combination with UTMD (15 mg/kg curcumin administered with 2 × 108 MBs/kg) rapidly produced PD-like syndrome alleviation and eventually reached a fully normal level after 4 treatments with CPC-UTMD. MPTP caused severe dopaminergic neuron loss in the striatum and treatment with CPC NPs with 5% PS 80 in combination with UTMD resulted in abundant DA and its metabolites. The dopaminergic neuron recovery efficiency in substantia nigra pars compacta after treatment with CPC NPs with 5% PS 80 in combination with UTMD was histologically confirmed.	Zhang et al., 2018
Liposomal-formulated curcumin (Lipocurc) obtained from SignPath Pharma Inc., Pennsylvania, USA	Park 7 (DJ-1)-gene knockout rat model	Lipocurc treatment of DJ-1-KO rats 20 mg/kg given i.v. 3 times/d for 8 wk improved the motor behavior impairment to a greater extent than PBS treatment of DJ-1-KO rats. Lipocurc significantly blocked neuronal apoptosis in striatum with the apoptotic index of treated DJ-1-KO rats being markedly reduced compared to DJ-1-KO rats treated with PBS. There was preliminary evidence that Lipocurc stimulated dopamine neurons in the substantia nigra, with ratio of immature to mature dopamine neurons in substantia nigra being statistically higher in treated DJ-1-KO rats compared to DJ-1-KO rats treated with PBS.	Chiu et al., 2013
**Huntington’s disease**			
Solid lipid curcumin particles (SLCPs) were suspended in PBS and delivered by oral gavage every other day for 8 wk.	YAC128 transgenic mice, 11 mon.	On the active avoidance task, there was a significant difference in latency to escape between WT and YAC128 mice, indicating that YAC128 mice have learning and memory deficits. SLCP treatment 100 mg/kg prevented learning and memory deficits in YAC128 mice. YAC128 mice treated with SLPs also showed a significant decrease in the latency to escape compared to WT and YAC128 mice, indicating that SLP treatment also improved memory and learning in YAC128 mice. There was a greater total length of dendrites of medium spiny neurons in vehicle-treated WT mice, compared to vehicle-treated YAC128 mice and SLP-treated mice 100 mg/kg. However, there were no significant differences between WT-vehicle mice and YAC128 mice treated with SLCPs. The dendrites in YAC128 mice treated with SLCPs were longer compared to YAC-vehicle controls, suggesting that SLCP treatment preserved/increased the total length of dendrites. There were also significant increases in the number of dendrites in YAC128 mice treated with SLCP compared to YAC128-vehicle mice, suggesting that SLCPs treatment increased or preserved the number of dendrites in YAC128 mice.	Gharaibeh et al., 2020
Curcumin encapsulated solid lipid nanoparticles (C-SLNs) administered by gavage at 1 h after 3-NP injection for 7 d. Average particle size 148 nm.	Wistar rats, female, 180–200 g, injected i.p. with 3-NP 20 mg/kg in saline for 3 d.	Using an actophotometer, administration of C-SLNs 40 mg/kg/day resulted in 20% increase in locomotor activity of 3-NP-treated animals. C-SLNs administration resulted in 37% decrease in total time of 3-NP-treated animals to traverse the narrow beam, and average velocity increased by 58% after C-SLNs administration.	Sandhir et al., 2014
		C-SLN-treated animals showed significant increase in the activity of mitochondrial complexes and cytochrome levels. C-SLNs also restored the GSH levels and SOD activity. Moreover, significant reduction in mitochondrial swelling, lipid peroxidation, protein carbonyls and ROS was observed in rats treated with C-SLNs.	
**Multiple sclerosis (demyelination stage)**			
Curcumin loaded self-nanoemulsifying drug delivery system (SNEDDS), with and without piperine in black seed oil, and given i.p. daily for 7 wk. Droplet size of curcumin and piperine loaded SNEDDS was 51 nm, and of drug-free SNEDDs was 28 nm.	Swiss albino mice, male, 21–25 g, fed 0.2% w/w CPZ-mixed chow daily for 5 wk to induce demyelination.	At the end of the demyelination stage (wk 5), mice from the CPZ group and other treatment groups spent shorter durations in the novel arm compared to the time spent in the same arm by mice of the control group. The analysis of novelty preference suggested that mice of CPZ group had significantly reduced preference for novel objects compared to the control group at wk 5. The mice in the treatment groups showed a better preference for novel objects compared to the mice from CPZ group. At wk 5, the SOD, GSH and GPX levels in the hippocampus of CPZ group were significantly lower than in the control group, whereas the levels in the curcumin 10 mg/kg nanoformulation groups were significantly increased compared to CPZ group. The TNF-α, NGF and COX-2 levels were significantly increased in CPZ group compared to the control group; however, the curcumin nanoformulation groups had lower levels of TNF-α, NGF and COX-2 than the CPZ group at wk 5. The examination of brain sections from CPZ group at 5 wks revealed a marked reduction in the intensity of LFB staining of corpus callosum compared to the control group and there were many areas of demyelination, fragmentation, and disorganization of corpus callosum nerve fibers. In curcumin + piperine group, there were predominantly myelinated nerve fibers, which appeared more or less similar to the control group. Oligodendrocytes appeared preserved and arranged in between the nerve fibers.	Alam et al., 2024

Aβ: Amyloid-β; CA: curcumin analogue; CAT: catalase; CN: curcumin nanoparticle; COX-2: cyclooxygenase-2; CPC NPs: curcumin-loaded polysorbate 80-modified cerasome nanoparticles; CPZ: cuprizone; CSF: cerebrospinal fluid; C-SLNs: curcumin encapsulated solid lipid nanoparticles; CUR: curcumin; CUR-PLGA-NP: curcumin-poly-lactic-co-glycolic acid-nanoparticle; DA: dopamine; DG: dentate gyrus; EXOs-CUR: curcumin loaded exosomes; GPX: glutathione peroxidase; GSH: glutathione; hEnSCs: human endometrial stem cells; i.c.v.: intracerebroventricular; IFN-γ: interferon-γ; IL-1β: interleukin-1β; IL-6: interleukin-6; i.p.: intraperitoneal; i.v.: intravenous; LCN: lipid core nanocapsules; LFB: Luxol Fast Blue; MDA: malondialdehyde; MEL: meloxicam; MPTP: 1-methyl-4-phenyl-1,2,3,6-tetrahydropyridine; NanoCA: nanoformulation of curcumin analogue; NC: curcumin nanoparticles; NCF: novel curcumin formulation; NGF: nerve growth factor; NLC C: curcumin liquid core nanocapsules; NO: nitric oxide; 3-NP: 3-nitropropionic acid; OA: okadaic acid; OB: olfactory bulb; 6-OHDA: 6-hydroxydopamine; PBS: phosphate-buffered saline; PD: Parkinson’s disease; PIP: piperine; p.o.: per oral; ROS: reactive oxygen species; SLCPs: solid lipid curcumin particles; SNEDDS: self-nanoemulsifying drug delivery system; SNpc: substantia nigra pars compacta; SOD: superoxide dismutase; S-SNEDDS: solid self-nanoemulsifying drug delivery system; STZ: streptozotocin; TH: tyrosine hydroxylase; TNF-α: tumor necrosis factor-α; UTMD: ultrasound-targeted microbubble destruction; wk: week; WT: wild type.

### Curcumin nanoforms to treat animal models of neurodegenerative disease

#### Alzheimer’s disease

Various animal models of AD were used and these included mice treated with aluminium chloride (AlCl_3_), Aβ peptide (Aβ), or okadaic acid, and rats treated with streptozotocin or Aβ. PPSwe/PS1deE9 transgenic mice and Tg2576 transgenic mice were used. These are all well-established model systems for the study of AD (for behavioral and neuronal changes, see Xu et al., 2023).

Ahmad and Hafeez (2023) prepared CUR-PIP solid self-nanoemulsifying drug delivery system (S-SNEDDS) nanoformulations with curcumin loading efficiency of 87%–99% and piperine loading efficiency of 91%–99%. Swiss albino mice, adult males, 18–22 g, were divided into five groups (*n* = 6 per group). Group 1 (vehicle control): mice received normal saline 2.5 mL/kg. Group 2 (negative control/AD): mice received AlCl_3_ 20 mg/kg intragastrically + D-galactose 120 mg/kg intraperitoneal (i.p.) in normal saline. Group 3 (treatment dose I): after AD inducer (AlCl_3_ + D-gal), mice received S-SNEDDS containing 25 mg/kg curcumin + 5 mg/kg piperine in normal saline. Group 4 (treatment dose II): after AD inducer (AlCl_3_ + D-gal), mice received S-SNEDDS containing 50 mg/kg curcumin + 10 mg/kg piperine in normal saline. Group 5 (donepexil): after AD inducer (AlCl_3_ + D-gal), mice received donepexil 1.0 mg/kg in normal saline. All treatments were dissolved in normal saline 2.5 mL/kg and administered to mice through oral gavage for 21 days. At the end of treatments, from the next day behavioral or learning tests were started. The total duration of the study was 12 weeks.

Curcumin nanoparticle (CN) purchased from One Planet Nutrition Company, Florida, USA, was used to treat rats by Noor et al. (2022). Wistar albino rats, adult males, 250–350 g, were divided into three groups (*n* = 10 per group, 8 for biochemical analysis and 2 for histopathology). Group 1 (control): rats received daily oral administration of distilled water for 6 days; on day 7 of administration, animals received a single bilateral intracerebroventricular (i.c.v.) injection of artificial cerebrospinal fluid (CSF); the oral administration of distilled water was continued for 8 days. Group 2 (AD model): rats received daily oral administration of distilled water for 6 days; on day 7 of administration, animals received a single bilateral i.c.v. injection of streptozotocin (3 mg/kg in artificial CSF) to induce AD; the oral administration of distilled water was continued for 8 days. Group 3 (CNs): rats received daily oral administration of CNs (50 mg/kg in distilled water) for 6 days; on day 7 of administration, animals received a single bilateral i.c.v. injection of streptozotocin (3 mg/kg in artificial CSF) to induce AD; the oral administration of distilled water was continued for 8 days. On day 8 post induction of AD, the Morris water maze test was performed to test the spatial memory of animals. Each animal was sacrificed by decapitation and the brain was rapidly removed and the cortex and hippocampus dissected.

Lipid core nanocapsules (LCN) loaded with curcumin and meloxicam were prepared by Gutierrez et al. (2021). Swiss mice, male, 40–50 g, 3 months old, were divided into eight groups (*n* = 8–9 per group). Control group (*n* = 9): sterile water (3 µL, i.c.v.) and unloaded LCN (LCN U) (17 mL/kg, gavage). Aβ group (*n* = 9): Aβ (3 nmol/3 µL, i.c.v.) and unloaded LCN U (17 mL/kg, gavage). Aβ + LCN CUR (*n* = 8): Aβ (3 nmol/3 µL, i.c.v.) and curcumin-loaded LCN (10 mg/kg, 17 mL/kg, gavage). Aβ + LCN MEL (*n* = 8): Aβ (3 nmol/3 µL, i.c.v.) and meloxicam-loaded LCN (5 mg/kg, 17 mL/kg, gavage). Aβ + LCN MEL-CUR (*n* = 9): Aβ (3 nmol/3 µL, i.c.v.) and meloxicam + curcumin co-loaded LCN (5 and 10 mg/kg, respectively, 17 mL/kg, gavage). Aβ + CUR (*n* = 8): Aβ (3 nmol/3 µL, i.c.v.) and curcumin (10 mg/kg in canola oil, 17 mL/kg, gavage). Aβ + MEL (*n* = 8): Aβ (3 nmol/3 µL, i.c.v.) and meloxicam (5 mg/kg in canola oil, 17 mL/kg, gavage). Aβ + MEL-CUR (*n* = 8): Aβ (3 nmol/3 µL, i.c.v.) and meloxicam + curcumin (5 and 10 mg/kg, respectively, in canola oil, 17 mL/kg, gavage). Treatments were started 24 hours after infusion of Aβ or vehicle, and mice were treated by intragastric administration on days 2, 4, 6, 8, 10, and 12. From the sixth day, mice were subjected to behavioral tests. At 24 hours after the last drug administration, mice were euthanized, and the prefrontal cortex and hippocampus were dissected. Long-term memory was measured by the object recognition test.

Wang et al. (2019) prepared curcumin-primed exosomes (Exo-CUR). Macrophage RAW264.7 cells were treated with curcumin (40 µg/mL) for 24 hours. The culture medium was collected and centrifuged (3000 rpm, 10 minutes) and the supernatant was collected. The supernatant was ultracentrifuged (100,000 rpm, 1 hour) to concentrate Exo-CUR into a pellet. C57BL/6 mice, 5–7 weeks, were divided into five groups (*n* = 3 per group). Okadaic acid (OA) was used to induce tau hyperphosphorylation by injecting 2 µL of OA (0.2 µM) in artificial CSF into the right dorsal hippocampus. Group 1 (sham group): mice in the normal control group received daily injection i.p. of 0.2 mL saline for 7 consecutive days. Group 2 (OA group): OA-treated mice received daily injection i.p. of 0.2 mL saline for 7 consecutive days. Group 3 (OA + CUR): OA-treated mice received daily injection i.p. of 0.2 mL of curcumin (100 µg/mL) in saline suspension for 7 consecutive days. Group 4 (OA + Exo-CUR): OA-treated mice received daily injection i.p. of 0.2 mL of Exo-CUR (100 µg/mL of curcumin) in saline suspension for 7 consecutive days. Group 5 (OA + Exo): OA-treated mice received daily injection i.p. of 0.2 mL of Exo in saline suspension for 7 consecutive days. The memory and spatial learning abilities of the mice were examined by the Morris water maze test.

Curcumin-loaded nanostructured lipid carriers (CUR-NLCs) and unloaded NLCs were prepared by Sadegh Malvajerd et al. (2019). Sprague-Dawley rats, male, 200–220 g, 6–8 weeks, were randomly divided into five groups (*n* = 7 per group). Sham: saline intravenous (i.v.) 1 mL/d for 4 days. Aβ AD control: saline i.v. 1 mL/d for 4 days. Unloaded NLCs: blank NLCs i.v. 1 mL/d for 4 days. CUR-NLCs + Aβ (Prevention): CUR-NLCs i.v. 1 mL/d (4 mg/kg/d dose of curcumin) for 4 days before Aβ injections. Aβ + CUR-NLCs (Treatment): CUR-NLCs i.v. 1 mL/d (4 mg/kg/d dose of curcumin) for 4 days after Aβ injections in training days. Morris water maze tests were performed from the 17^th^ day of administration. Following the Morris water maze test, rats were euthanized, and brain hippocampal tissue was extracted and kept at –80 °C until biochemical studies were performed. Histopathological studies were performed on 3 rats in each group. To create the AD model, 2 µL of Aβ suspension (100 ng/µL) was injected on each side of the posterior hippocampus.

Giacomeli et al. (2019) prepared curcumin liquid core nanocapsules (LNC) that were given orally. Swiss albino mice, 30–35 g, 18–22 months, were randomly divided into eight groups (*n* = 8 per group). Mice were treated for 14 days after receiving i.c.v. injection of Aβ_1–42_ 400 pmol (3 µL). Group 1: Saline/Sham. Group 2: Free curcumin 50 mg/kg (FC50)/Sham. Group 3: curcumin loaded-lipid core nanocapsules 10 mg/kg (LNC10)/Sham. Group 4: curcumin loaded-lipid core nanocapsules 1 mg/kg (LNC1)/Sham. Group 5: Saline/Aβ_1–42_. Group 6: FC50/Aβ_1–42_. Group 7: LNC10/Aβ_1–42_. Group 8: LNC1/Aβ_1–42_. Mice were treated for 14 days after i.c.v. injection of Aβ_1–42_ or vehicle. On day 15 after i.c.v. injection, mice underwent behavioral assessment and were euthanized. Blood, prefrontal cortex, and hippocampus were obtained for assay. Spatial memory was measured in a probe test 24 hours after the last training trial in the Morris water maze test.

Curcumin liquid core nanocapsules (NLC C) were prepared by Fidelis et al. (2019). Swiss albino mice, male, 40–50 g, 3 months old, were randomly divided into five groups (*n* = 7–8 per group). Group 1 (Control): vehicle i.c.v. and NLC U given by intragastric route (i.g.). Group 2 (Aβ): Aβ_25–35_ i.c.v. and NLC U i.g. Group 3 (NLC C): vehicle i.c.v. and NLC C i.g. Group 4 (Aβ+NLC C): Aβ_25–35_ i.c.v. and NLC C i.g. Group 5 (Aβ+C): Aβ_25–35_ i.c.v. and free curcumin in canola oil i.g. Vehicle was sterile water 3 µL. Treatments with NCL U (10 mL/kg), NCL C (10 mg/mL) and free curcumin in canola oil (10 mg/mL) were carried out by i.g. once every 48 hours i.e., days 2, 4, 6, 8, 10, and 12. On day 12 after Aβ_25–35_ administration, open field test, tail suspension test, and forced swimming test were performed 30 minutes after the last treatment. On the following day, mice were decapitated, and the prefrontal cortex and hippocampus were dissected for analyses. Aβ_25–35_ was administered i.c.v. 3 nmol in 3 µL.

Parikh et al. (2018) developed a novel CUR formulation (NCF). APPSwe/PS1deE9 transgenic mice, aged 14 months, were randomly divided into six groups (*n* = 9–12 per group). Group 1 (basal control, no treatment): mice underwent behavioral tests at 14 months of age. Group 2 (sham control, no treatment): mice were administered normal drinking water for 3 months. Group 3 (blank control): mice were supplied with blank formulation containing Soluplus (equivalent amount to that used in NCF) in drinking water for 3 months. Group 4 (CUR control): mice were administered with a mixture of CUR and Soluplus in drinking water for 3 months (equivalent to the amount used in NCF). Group 5 (NCF): mice were administered NCF in drinking water for 3 months (equivalent to CUR dose 47 mg/kg). Group 6 (donepezil): administered donepexil in drinking water for 3 months (equivalent to donepexil dose 2 mg/kg). One group of C57BL/6 mice as a control for the APPSwe/PS1deE9 strain was supplied with normal drinking water. Behavioral tests were performed at 17 months of age of APPSwe/PS1deE9 (transgenic, Tg) and C57BL/6 mice (wild-type, WT) after 3 months of treatment except for the one group of APPSwe/PS1deE9 (14-month Tg Control), which was performed at 14 months of age.

Curcumin-PLGA-nanoparticles (CUR-PLGA-NPs) were prepared by Tiwari et al. (2014). Rats at post-natal day 28 received an injection of 2 µL Aβ_1–42_ (2 µg/µL in saline) into both sides of the hippocampus. After 1 week, at post-natal day 35, rats were treated with bulk curcumin and CUR-PLGA-NPs daily for 2 consecutive weeks. Experimental groups (*n* = 6 rats per group) were as follows. Group 1 (sham): received 2 µL of normal saline as vehicle. Group 2: intrahippocampal injection of Aβ in saline. Group 3: Aβ + empty PLGA-NPs. Group 4: Aβ + bulk curcumin (0.5 mg/kg body weight, i.p.). Group 5: Aβ + CUR-PLGA-NPs (0.5 mg/kg body weight, i.p.). Group 6: Aβ + bulk curcumin (20 mg/kg body weight, i.p.). Group 7: Aβ + CUR-PLGA-NPs (20 mg/kg body weight, i.p.). A set of rats was sacrificed at post-natal day 49 for immunohistochemistry, and another at post-natal day 70 for cell survival/neuronal differentiation in the dentate gyrus and subventricular zone. The learning and memory ability of the control and curcumin-treated rats was measured following the assessment of two-way conditioned avoidance response behavior in a shuttle box (Mishra et al., 2012).

Cheng et al. (2013) examined the effect of curcumin nanoparticles (NC) in Tg2576 transgenic mice. Tg2576 mice, 9 months, were divided into three groups (*n* = 20 per group): for NC, 6 males/14 females; for curcumin, 4 males/16 females; for controls, 5 males/15 females. All mice were fed with 23 mg/kg (curcumin/body weight) NC, curcumin, or water by gavage. Mice were treated once every week for 3 months. The radial arm maze test was used for evaluating spatial learning and memory. Contextual fear conditioning was used to assess memory of an unconditioned stimulus such as a foot shock with a conditioned stimulus such as a particular cage or tone.

#### Parkinson’s disease

The model systems used were mice treated with the neurotoxins 6-hydroxydopamine (6-OHDA) or 1-methyl-4-phenyl-1,2,3,6-tetrahydropyridine (MPTP), which have been employed in a previous study (for behavioral and neuronal changes, see Khan et al., 2023). DJ-1 knockout (KO) rats were also used and have impairments and progressive neurodegeneration in the substantia nigra region of the brain (Dovonou et al., 2023).

Mobahat et al. (2023) studied the effect of curcumin-loaded human endometrial stem cells derived exosomes (hEnSCs EXOs-CUR). C57BL/6 mice, male, 8 weeks, 27–32 g, were anesthetized. 10 µg 6-OHDA in saline with 0.02% ascorbic acid was injected into the striatum at 1 µL/min. Animals were divided into five groups (*n* = 5 per group) and all treatments were performed intranasally 10 times every other day with a defined dose for 28 days. Group 1 (control): healthy mice. Group 2: C57BL/6 mice (*n* = 4) were injected with 6-OHDA and treated with phosphate-buffered saline (PBS). Group 3: injected intranasally with 10 mg/kg body weight hEnSCs EXOs-CUR. Group 4: injected intranasally with free curcumin 10 mg/kg body weight. Group 5: injected intranasally with 10 µg naïve hEnSCs EXOs (1 mg/mL) dispersed in PBS as a carrier. At the end of the study on day 29, before sacrifice of mice, the rotarod test was applied to monitor motor behavior. Expression of α-synuclein aggregation and dopaminergic neurons in brain tissue sections was investigated by immunohistochemistry.

Curcumin analog nano (NanoCA) was prepared by Liu et al. (2020b). C57BL/6 mice, male, approximately 22 g, were injected i.p. with MPTP-HCl (30 mg/kg) daily for 7 consecutive days. Intranasal administration of saline or NanoCA (5 mg/kg) was performed 7 times during 10 days (days –2, –1, 0, 2, 4, 6, 8, with day 0 being the day of starting MPTP injections). Mice were monitored for 3 weeks to evaluate behavior and ensure lesion stabilization before sacrifice. NanoCA 5 mg/kg (containing 1.35 mg/kg CA in 20 µL saline) was administered to 6 mice by rapid arousal intranasal delivery system. Tissues including brain olfactory bulb, cerebrum (except for olfactory bulb), cerebrospinal fluid, and plasma were collected to determine CA content after 24 hours.

Curcumin-loaded polysorbate 80-modified cerasome nanoparticles (CPC NPs) were prepared by Zhang et al. (2018). C57BL/6 mice were treated by repeated injections i.p. of MPTP (40 mg/kg) solution for 7 consecutive days. The mice were divided into five groups. Group 1 (control); Group 2: only curcumin-loaded cerasomes with no polysorbate 80; Group 3: only CPC with 5% polysorbate 80; Group 4: 5% polysorbate 80 modified cerasomes with no curcumin in combination with ultrasound-targeted microbubble destruction; Group 5: CPC with 5% polysorbate 80 in combination with ultrasound-targeted microbubble destruction (15 mg/kg curcumin was administered with 2 × 10^8^ microbubbles [MBs]/kg). The mice were treated every 2 days for 4 times to ensure effective drug accumulation in the brain. For the groups with ultrasound-targeted microbubble destruction application, the mice were depilated, and the ultrasonic probe was positioned over the corpus striatum. Ultrasound (1.3 MHz) was performed for 60 seconds on both sides of the brain after different i.v. administrations. Behavioral tests reflected the recovery status of mice with different treatments (*n* = 10 per group).

Chiu et al. (2013) obtained liposomal-formulated curcumin (Lipocurc) from SignPath Pharma Inc., Pennsylvania, USA. DJ-1 KO rats were randomly assigned to the following groups: Group 1 (*n* = 4): DJ-1-KO-Lipocurc received 20 mg/kg Lipocurc i.v.; Group 2 (*n* = 4): DJ-1 KO treated with PBS i.v.; Group 3 (control, *n* = 4): DJ-1 WT (Long-Evans rats) received PBS i.v. All animals were injected through the tail vein 3 times daily for 8 weeks. Injection volume was 1 mL/kg for each rat (this measure was adjusted on each day of injection for weight change). On day 3 prior to injections, each rat was tested on the catalepsy test, followed by the rotarod test to examine the motor integrity of the rats. Each rat completed two trials per session for the duration of the 8 weeks. During the last week of injections, all rats were assessed on several behavioral tests (including open field test and cylinder test). At 72 hours after the last injection, the rats were euthanized and the brains were removed. Tissue sections were prepared for immunostaining for TUNEL assays and tyrosine hydroxylase labeling assays of dopamine neurons in the striatum.

#### Huntington’s disease

The animal models used were YAC128 transgenic mice and rats treated with the neurotoxin 3-nitropropionic acid (3-NP). These are well-researched animal models of HD (for behavioral and neuronal changes, see Nittari et al., 2023).

Gharaibeh et al. (2020) studied the effects of solid lipid curcumin particles (SLCPs). YAC128 and WT mice, 11 months of age, were assigned to the following groups (6 males and 3 females per group): WT vehicle controls (WT + PBS, *n* = 9); YAC128 vehicle controls (HD + PBS, *n* = 9); WT treated with SLCPs (WT + SLCP, *n* = 9); YAC128 treated with SLCPs (HD + SLCPs, *n* = 9); WT treated with solid lipid particles (SLPs) (WT + SLP, *n* = 9); YAC128 treated with SLPs (HD + SLP, *n* = 9). The appropriate dose of SLCPs or SLPs (100 mg/kg) for every mouse was suspended in PBS and administered by oral gavage. The gavage treatments were done in the morning on every other day for 8 weeks. Vehicle controls were treated with PBS (an equal volume to that used in SLCP dosing). The active avoidance test was performed for all mice, with acclimatization done at the beginning of week 8 of treatments. Three mice (2 males and 1 female) from each group were randomly selected, euthanized, and the brains were removed for Golgi-Cox staining. For protein analyses, three mice (2 males and 1 female) from each group were randomly selected, euthanized and the brains were removed. To test the differences in learning on the active avoidance task, a non-aversive stimulus (tone) immediately preceded an aversive stimulus (shock) and the ability of the mice to escape the aversive stimulus was measured.

Curcumin encapsulated solid lipid nanoparticles (C-SLNs) were prepared by Sandhir et al. (2014). Wistar rats, female, 180–200 g, were randomly assigned to the following four groups (*n* = 5 per group). Control (vehicle): animals were given saline i.p.; Control + C-SLN: C-SLN was administered to rats orally at a daily dose of 40 mg/kg body weight using gavage; 3-NP: HD was induced by administering 3-NP at a dosage of 20 mg/kg body weight i.p. dissolved in saline for 3 consecutive days; 3-NP + C-SLN: C-SLN was administered orally to 3-NP animals at 1 hour after 3-NP injection for 7 days at a daily dose of 40 mg/kg body weight using gavage. Motor coordination and gait analysis were performed. Animals were killed on day 7, the brains were removed, and the corpus striatum was dissected.

#### Multiple sclerosis (demyelination stage)

The animal model used was mice treated with cuprizone (CPZ), which has been extensively studied (for behavioral and neuronal changes, see Dedoni et al., 2023).

A curcumin loaded self-nanoemulsifying drug delivery system (SNEDDS) was prepared by Alam et al. (2024), which was a nanoformulation of curcumin with and without piperine in black seed oil (BSO). Swiss albino mice, male, 21–25 g, were randomly divided into 5 groups (15 mice per group). Group 1 (Control): received 0.1 mL saline i.p. and normal chow daily for 7 weeks; Group 2 (Cuprizone group): received 0.1 mL saline i.p. and CPZ-mixed chow daily for 5 weeks; Group 3: received 0.1 mL BSO-based blank formulation i.p. and CPZ-mixed chow daily for 5 weeks; Group 4: received a BSO-based curcumin formulation 0.1 mL i.p. and CPZ-mixed chow daily for 5 weeks; Group 5: received a BSO-based nanoformulation of curcumin with piperine i.p. and CPZ-mixed chow daily for 5 weeks. At the end of the demyelination stage (week 5), cuprizone feeding was stopped in all groups, but treatment with saline and different nanoformulations continued until the end of week 7. At the end of week 5, half of the mice in each group were randomly selected and euthanized. The whole brain was then removed. The collected brains were dissected to obtain specific regions of the brain, including the hippocampus, frontal cortex, cerebral cortex, brain stem, and cerebellum. The same procedure was followed for the remaining mice at the end of week 7. A dosage of curcumin (10 mg/kg) was selected. Cuprizone feeding for 5 weeks resulted in a significant decrease in short-term spatial working memory as compared to the short-term spatial working memory in mice of the control group.

### Quercetin nanoforms to treat animal models of neurodegenerative disease

#### Alzheimer’s disease

The animal models used were rats treated with aluminium chloride or streptozotocin. Also, APP/PS1 transgenic mice and senescence-accelerated mouse-prone 8 (SAMP8) mice were used. The latter is a model of age-related cognition decline (for neuronal changes, see Liu et al., 2020a).

Mobasheri et al. (2023) prepared magnetic quercetin-neuropeptide nanoparticles. Streptozotocin was used to model AD in adult female rats, average weight of 250 g. The following treatments were applied to AD rats for 7 days: (A) AD Control (without treatment); (B) Treatment of 1 mL nano quercetin 5 ng/d; (C) Treatment of 2 mL nano quercetin 10 ng/d; (D) 2 mL nano neuropeptide quercetin treatment 10 ng/d; (E) healthy control group in 3 repetitions. After 7 days, the rats were assessed using behavioral tests.

Quercetin nanoemulsion particles were studied for effect in adult albino rats, male, 140 ± 10 g, given AlCl_3_ (100 mg/kg/d) orally in saline for 30 days by Alaqeel et al. (2022). Rats were divided into six groups (*n* = 8 per group). Control group: received vehicle. Quercetin group: received i.p. injection of quercetin 15 mg/kg/d; AD group: received AlCl_3_ orally; Treated group 1: received AlCl_3_ orally and during this period animals received i.p. injection of quercetin free nanoemulsion 15 mg/kg/d; Treated group 2: received AlCl_3_ orally and during this period animals received quercetin orally 15 mg/kg/d; Treated group 3: received AlCl_3_ orally and during this period animals received i.p. injection of quercetin nanoemulsion 15 mg/kg/d. At the end of the experimental period (30 days), rats were fasted overnight, anesthetized, blood collected from the inferior vena cava, and the brains were quickly removed and divided into two parts (the first part for biochemical analysis and the second part for immunohistochemistry).

Amanzadeh Jajin et al. (2021) prepared quercetin-conjugated superparamagnetic iron oxide nanoparticles (QT-SPIONs). AlCl_3_ (100 mg/kg/d) was given orally in distilled water for 42 days to Wistar rats, male, 7 weeks, 180 ± 20 g. Rats were randomly divided into six groups (*n* = 8 per group). Group 1 (control): received no treatment; Group 2 (sham): received 1 mL distilled water each day; Group 3 (AD group): received AlCl_3_ 100 mg/kg/d in 1 mL distilled water; Group 4: received AlCl_3_ 100 mg/kg/d and SPION 25 mg/kg/d in 1 mL distilled water; Group 5: received AlCl_3_ 100 mg/kg/d and QT 25 mg/kg/d in 1 mL distilled water; Group 6: received AlCl_3_ 100 mg/kg/d and QT-SPION 25 mg/kg/d in 1 mL distilled water. All administrations were performed by oral administration (gavage) for 42 continuous days. Spatial learning and memory changes were assessed using the Morris water maze. Rats were trained for 5 days as the acquisition phase between the 17^th^ and 20^th^ day of treatment. Probe trials were performed twice on the 21^st^ and 42^nd^ day of treatment. For this test, the invisible platform was removed. The passive avoidance test was used to assess fear-induced learning and memory changes in studied rats. Training was performed once on the 19^th^ day of treatment. At the end of the study, animals were sacrificed and the brains were removed.

Quercetin nanoparticles (QNPs) were produced by Rifaai et al. (2020). Sprague–Dawley rats, male, 6–8 weeks, 150–200 g, were divided randomly into four groups (*n* = 6 per group). Control group: received saline; AD group: received AlCl_3_ 100 mg/kg/d; QNPs prophylactic group: received AlCl_3_ 100 mg/kg/d + QNPs 30 mg/kg/d; QNPs treatment group: received QNPs 30 mg/kg/d after induction of AD. At the end of the study, animals were sacrificed, brains removed, and hippocampi dissected. QNPs were given intragastrically.

Liu et al. (2020c) studied the effect of quercetin-modified sulfur nanoparticles embedded in microbubbles (Qc@SNPs-MB) in APP/PS1 AD mice, at 30 weeks of age. Mice were randomly divided into four groups (*n* = 5 per group) and injected with quercetin (5 mg/kg), SNPs-MB (5 mg/kg), Qc@SNPs-MB (NPs 5 mg/kg), and an equal volume of saline via their tail vein, respectively. SNPs-MB and Qc@SNPs-MB were subjected to ultrasound exposure for 10 minutes after injection (sound pressure approximately 1000 kPa). Treatment was performed weekly (Monday and Thursday) for 5 weeks for a total of 10 injections. After 5 weeks of treatment, the Morris water maze test was used to assess the learning and cognitive ability of the AD mice.

Quercetin-loaded nanoparticles were prepared by Moreno et al. (2017) and studied for effect in SAMP8 and SAMR1 mice, male, 28–30 g, 5 months of age. Four groups of animals were used (*n* = 8 mice per group). The first group of SAMP8 mice received by the oral route a daily dose of quercetin as an oral solution (quercetin, 25 mg/kg). The second group of SAMP8 mice received orally, every two days, a dose of 25 mg/kg of quercetin-loaded nanoparticles. In both cases, the animals were treated for 2 consecutive months. Behavioral tests were begun at 4 weeks after the start of quercetin administration and extended throughout the assays. The last dose for animals treated with free quercetin and quercetin-loaded nanoparticles was administered 24 and 48 hours before their sacrifice, respectively. As controls, two groups of animals were used. The first group included SAMP8 mice whereas the second one was of SAMR1 mice. In both cases, the animals received orally 1 mL of saline every day. Mice were sacrificed on day 60 and the hippocampus of brains was removed for analysis of glial fibrillary acidic protein and CD11b.

#### Huntington’s disease

Debnath et al. (2019) examined the effects of quercetin encapsulated polymer nanoparticles on cultured HD150Q cells, a HD model cell line.

## Discussion

At present, the inability of therapeutic drugs and biomolecules to effectively cross the blood–brain barrier makes treating neurodegenerative diseases difficult (Pardridge, 2020). The passage of biomacromolecules and low molecular weight compounds from the blood to the brain is restricted by the high concentrations of drug-metabolizing enzymes and the expression of outward drug efflux transporters, together with the presence of tight junctions between endothelial cells in the blood vessels supplying the brain (Kabanov and Gendelman, 2007). Nanoparticle delivery vehicles of 1–100 nm in size can penetrate significant physiological barriers such as those in the lungs, liver, gastrointestinal tract, blood vessels, tumor vasculature, mucosal membranes, and the blood–brain barrier. The flavonoids curcumin and quercetin have several important properties such as anti-inflammatory and antioxidant activity, but their use in treating neurodegenerative diseases is limited by low aqueous solubility, low bioavailability, rapid metabolism, and low transport across the blood–brain barrier. However, these disadvantages can be overcome by using nanocarriers to deliver curcumin and quercetin.

In the studies reviewed many different nanoformulations of curcumin and quercetin had been prepared as particles or emulsions. For nanocurcumin, particle sizes ranged from 30–312 nm and emulsion droplet sizes from 22–310 nm (**Table 1**), and for nanoquercetin, particle sizes were 29–750 nm (**Table 2**). For both nanocurcumin and nanoquercetin, most of the studies had been performed with mouse and rat models of AD (mostly males), which included the use of aluminium chloride given intragastically, streptozotocin administered i.c.v., aggregated Aβ given i.c.v., okadaic acid injected into the hippocampus, transgenic models such as APPswe/PS1deE9 mice and Tg2576 mice, and SAMP8 (senescence accelerated) inbred mice. Most of these studies had been performed with young adult animals; however, five studies utilized aged mice (Cheng et al., 2013; Moreno et al, 2017; Parikh et al., 2018; Giacomeli et al., 2019; Liu et al., 2020c).

**Table 2:** Effects of quercetin nanoformulations in animal models of neurodegenerative disease

Quercetin nanoformulation and administration	Animal model of neurodegenerative disease	Behavioral and neurorestorative effects of quercetin nanoformulations in animal models of neurodegenerative disease	Reference
**AD**			
Magnetic quercetin-neuropeptide nanoparticles (MQNPN) for 7 d; it was not reported how the nanotreatment was administered. MQNPN size 29 nm.	Adult rats, female, average wt 250 g. STZ was used to model AD in rats (Salkovic-Petrisic et al., 2013), but details not reported.	Histological studies of the hippocampus in rats before and after treatment showed neuronal eosinophilia in the hippocampus of AD rats was increased, but that of treated rats was close to that of healthy rats. Quercetin 5 or 10 ng/d had high antioxidant activity in the AD rat samples compared to the control group (untreated AD rats). Changes in the expression of MAPT and APP genes in the brains of AD rats treated with MQNPN 10 ng/d were investigated using real-time PCR. The MAPT genes showed a significant decrease after treatment with MQNPN. Also, expression of APP gene decreased compared to AD rats, but did not reach the expression level of a normal rat. However, the APP gene also showed reduced expression at the control level in treatment with MQNPN. Both nano quercetin treatment and nano quercetin neuropeptide margatoxin (MQNPN) increased SOD activity in the hippocampal brain samples compared to the AD control.	Mobasheri et al., 2023
Quercetin nanoemulsion (QCNE) given i.p. for 30 d.	Adult albino rats, male, 140 ± 10 g, that were given AlCl3 (100 mg/kg/d) orally in saline for 30 d (Chen et al., 2021).	Treatment of AD rats with QC orally 15 mg/kg/d significantly increased brain SOD and GSH, and decreased the oxidation parameters MDA and AOPP, compared to AD group. Treatment with QCNE 15 mg/kg/d had the best results compared to QC alone to become near the control group, especially in SOD and GSH levels. QC administration significantly decreased the inflammatory markers. IL-1β, TNF-α, and adiponectin, and treatment with QCNE had the best results to become near the control group, especially in TNF-α and adiponectin. Immunohistochemistry showed a positive reaction against COX-2 for the AD group, while treatment with QC and QCNE had a negative reaction against COX-2. The levels of the neurotransmitters (norepinephrine, dopamine, and serotonin) as indicators of the disturbance of brain function were increased significantly in the AD group compared to the control. QC significantly decreased the levels of the neurotransmitters compared to the AD group, although they were still significantly higher than the control. Administration of QCNE significantly reduced these levels compared to AD but they were not significantly changed compared to the control. QCNE significantly decreased the neurotransmitter levels compared to QC alone.	Alaqeel et al., 2022
Quercetin-conjugated superparamagnetic iron oxide nanoparticles	Wistar rats, male, 7 wk, 180 ± 20 g, given AlCl3 (100 mg/kg/d) orally in distilled water for 42 d.	In Morris water maze test, treatment with QT-SPION, SPION, and QT, each at 25 mg/kg/d with AlCl3 had smaller escape latency and improved spatial memory compared to AlCl3 group. In the passive avoidance test, a significant decrease was observed in retention latency (RL) in the AlCl3 group in comparison with the control group. The AlCl3 + QT and AlCl3 + SPION groups showed significantly higher step-through latency and RL on the 21st and 42nd day of treatment in comparison with AlCl3 group, while improvements in step-through latency and RL in these groups were lower than the control, sham, and AlCl3 + QT-SPION groups. Transcription levels of the GPX1 gene were significantly decreased in AlCl3 group compared to the control group. Increases were observed in the AlCl3 + QT-SPION group compared to the AlCl3 group, and were similar to the control group. A significant increase was observed in the AlCl3 + SPION and AlCl3 + QT groups in comparison with the AlCl3 group, while the levels for these groups were still significantly lower than those for the control group. The CAT gene had significantly lower expression levels in the AlCl3 group compared to the control. The AlCl3 + QT-SPION group showed significantly higher expression levels than the AlCl3 group and results obtained for AlCl3 + QT-SPION were similar to the control group. The AlCl3 + QT and AlCl3 + SPION groups revealed no significant increase in expression levels of CAT gene compared to the AlCl3 group. The expression levels of BCL2 as an antiapoptotic gene showed a significant decrease in the AlCl3 group compared to the control group. In the AlCl3 + QT-SPION group there was an increase compared to the AlCl3 group. BAX as a proapoptotic gene had a significantly higher expression level in the AlCl3 group compared to the control group, and a recovery in the AlCl3 + QT-SPION group which was similar to the control group.	Amanzadeh Jajin et al., 2021
(QT-SPIONs) given by oral gavage for 42 d. Nanoparticles size 30–50 nm.			
Quercetin nanoparticles (QNPs) given intragastrically for 42 d. Nanoparticles size 520–750 nm.	Sprague-Dawley rats, male, 6–8 wk, 150–200 g, given AlCl3 (100 mg/kg/d) in water intragastrically for 42 d.	In the QNPs-prophylactic group treated with 30 mg/kg/d, the overall effect was that QNPs preserved the normal cellular morphology of most of the cells of the CA1, CA3 and DG regions of the hippocampus in comparison to the AD group. In the QNPs-treatment group treated with 30 mg/kg/d after induction of AD, cells in the CA1 and DG showed normal cellular morphology; cells in CA3 showed the least improvement with many pyramidal cells exhibiting morphological changes and vacuolated neuropils as seen in the AD group. Thus, the detrimental effect of AlCl3 on hippocampal neurons was reversed in CA1 and DG and partially reversed in CA3 by the administration of QNPs. QNPs administration in QNPs-prophylactic and -treated groups significantly reduced the number of degenerated neurons compared to the AD group. QNPs effect as prophylactic was more profound than as therapeutic in reducing the number of degenerated neurons but did not reach significance (P = 0.060). QNPs administration significantly reduced the number of cells with NFTs both in the prophylactic and treated groups by comparison to the AD group. The effect of QNPs as prophylactic produced a profound effect which was significant compared to the treated group. QNPs as prophylactic almost blocked the effect of AlCl3 on the neurons which showed a nonsignificant difference in comparison to the control. QNPs administration in the prophylactic and treated groups significantly reduced amyloid plaques compared to the AD group. There was no significant difference between the prophylactic group and treated group. However, QNPs administration as a prophylactic almost blocked the development of amyloid plaques associated with AlCl3 administration, shown by it being nonsignificantly changed compared to the control group. QNPs-prophylactic group restored the strong TH immunoreactivity in the cell bodies and axons resembling the control group. Also, QNPs-treated group showed positive TH reaction in cell bodies as well as axons of pyramidal neurons.	Rifaai et al., 2020
Quercetin-modified sulfur nanoparticles embedded in microbubbles (Qc@SNPs-MB) given i.v. and subjected to ultrasound (US) exposure for 10 min after injection (sound pressure approximately 1000 kPa). Treatment performed weekly (Monday and Thursday) for 5 wk: a total of 10 injections.	APP/PS1 AD mice, 30 wk	In Morris water maze test, when the platform was removed, the APP/PS1 mice did not stay in the target interval purposefully or look for the location of the platform, indicating severe memory impairment in the APP/PS1 mice. After treatment with Qc@SNPs-MB (NPs 5 mg/kg) + US, the number of mice crossing the platform increased significantly, showing the best therapeutic effect. There was a significant difference in the Aβ deposition between the APP/PS1 mice and WT mice. However, when treated with Qc@SNPs-MB + US, the content of Aβ plaques in the brain of the mice significantly decreased, indicating that Qc@SNPs could effectively reduce the deposition of Aβ in the brain. SNPs 5 mg/kg also reduced Aβ deposition in the brain of the AD mice, but the effect was not as marked as that of Qc@SNPs, indicating that sulfur nanoparticles have the ability to inhibit amyloid deposition. After treatment with Qc@SNPs-MB + US, there was a significant increase in Nissl bodies indicating that Qc@SNPs were effective in reducing neuronal loss in the AD mice. After treatment with SNPs, the number of neurons also recovered, indicating that reducing amyloid deposition in the brain can reduce neuron loss to some extent.	Liu et al., 2020c
Quercetin-loaded nanoparticles (NPQ) given orally, every 2 d for 2 consecutive mon. Nanoparticles size 260 ± 8 nm.	SAMP8 mice, male, 28–30 g, 5 mon.	In the Morris water maze test, during the acquisition phase of the test, the time spent to reach the platform was significantly lower for SAMR1 mice (used as a normalizing control strain for the SAMP strain) than for SAMP8 mice. Over trials, SAMR1 reduced the time required to reach the platform. The latencies exhibited by the saline-treated SAMP8 group did not significantly decrease over trials, indicating the existence of learning deficits in these mice. However, SAMP8 mice treated with NPQ 25 mg/kg/d showed a marked improvement in their behavioral performance as their escape latencies (on d 4, 6, and 7) were significantly shorter than for SAMP8 mice treated with saline. No significant differences were found between saline and Q-treated 25 mg/kg/day SAMP8 mice. Upon removal of the platform, SAMP8 control mice spent significantly less time in the target quadrant than SAMR1 mice. On d 4, no significant differences were found among saline, Q or NPQ-treated SAMP8 mice. However, on d 8 (24 hours after the final trial on the learning test), post hoc analysis revealed that SAMP8 mice treated with NPQ spent more time in the target quadrant than saline-treated SAMP8 mice, whereas no differences were observed between saline or Q-treated SAMP8 mice. Two different markers of brain inflammation were analyzed. The astrocyte marker GFAP displayed a significant increase in the hippocampus of SAMP8 mice in comparison to the SAMR1 control strain. NPQ significantly reduced hippocampal GFAP expression whereas this effect was not observed in Q-treated SAMP8 mice. No difference was found for CD11b, a microglia activation marker.	Moreno et al., 2017
**Huntington’s disease**			
Quercetin encapsulated polymer nanoparticles, size 30 nm.	Only cell culture studies were performed mainly using HD150Q cells, a Huntington’s disease model cell line.	Nanoquercetin (with 1 μM quercetin) efficiently inhibited huntingtin aggregation whereas molecular quercetin at 50 µM was inefficient in inhibiting mutant huntingtin aggregation. Nanoquercetin (with 1 μM of quercetin) enhanced the cell viability to 95% as compared to 70% viability of untreated cell and 49% viability for 50 μM molecular quercetin treated cells.	Debnath et al., 2019

Aβ: Amyloid-β; AD: Alzheimer’s disease; AOPP: advanced oxidation protein products; COX-2: cyclooxygenase-2; DG: dentate gyrus; GFAP: glial fibrillary acidic protein; GSH: glutathione; IL-1β: interleukin-1β; i.p.: intraperitoneal; i.v.: intravenous; MDA: malondialdehyde; MQNPN: magnetic quercetin-neuropeptide nanoparticles; NPQ: quercetin loaded nanoparticles; PCR: polymerase chain reaction; p.o.: per oral; Q: quercetin; QC: quercetin; Qc@SNPs-MB: quercetin-modified sulfur nanoparticles embedded in microbubbles; QCNE: quercetin nanoemulsion; QNPs: quercetin nanoparticles; qRT-PCR: quantitative real time polymerase chain reaction; QT: quercetin; QT-SPIONs: quercetin-conjugated superparamagnetic iron oxide nanoparticles; RL: retention latency; SNP: sulfur nanoparticles; SOD: superoxide dismutase; SPION: superparamagnetic iron oxide nanoparticles; STZ: streptozotocin; TH: tyrosine hydroxylase; TNF-α: tumor necrosis factor-α; US: ultrasound; UTMD: ultrasound-targeted microbubble destruction; wk: week; WT: wild type.

Several of the curcumin nanoformulations brought about improvements in learning and memory in behavioral tests in animal models of AD (Cheng et al., 2013; Tiwari et al., 2014; Parikh et al., 2018; Giacomeli et al., 2019; Sadegh Malvajerd et al., 2019; Wang et al., 2019; Gutierrez et al., 2021; Noor et al., 2022; Ahmad and Hafeez, 2023). They were effective in reducing oxidative stress as shown by improving malondialdehyde, glutathione, nitric oxide, reactive oxygen species to control levels in the brain (Sadegh Malvajerd et al., 2019; Noor et al., 2022). The levels of inflammatory markers such as TNF-α, IL-1β, IL-6, interferon-γ, and nuclear factor kappa B (Giacomeli et al., 2019), and Aβ plaques and neurofibrillary tangles in the brain were decreased (Cheng et al., 2013; Sadegh Malvajerd et al., 2019; Noor et al., 2022). Nanoforms of curcumin brought about a decrease in apoptosis (Wang et al., 2019) and the number of degenerated neurons in the hippocampus was reduced (Tiwari et al., 2014; Wang et al., 2019). Furthermore, nanocurcumin formulations were tested in animal models of PD, including injection of 6-OHDA into the striatum of mice, i.p. injection of MPTP into mice, and DJ-1-KO rats. Motor behavior, gait, and memory were improved with nanocurcumin (Chiu et al., 2013; Zhang et al., 2018; Liu et al., 2020b; Mobahat et al., 2023). Nanocurcumin reduced α-synuclein in the midbrain (Liu et al., 2020b; Mobahat et al., 2023) and dopaminergic neurons in the striatum and substantia nigra were increased, consistent with an improvement in dopamine deficiency and a reduction in apoptosis (Chiu et al., 2013; Zhang et al., 2018; Liu et al., 2020b; Mobahat et al., 2023). HD models included rats injected i.p. with 3-nitropropionic acid and YAC transgenic mice. Nanocurcumin improved locomotor activity (Sandhir et al., 2014), memory and learning (Gharaibeh et al., 2020), and the number of dendrites of medium spiny neurons was increased (Gharaibeh et al., 2020). In addition, nanocurcumin decreased mitochondrial swelling, lipid peroxidation, and reactive oxygen species (Sandhir et al., 2014). In a mouse model of demyelination in multiple sclerosis brought about by feeding cuprizone in the chow, nanocurcumin formulations decreased oxidative stress and inflammation (Alam et al., 2024). A nanoformulation of curcumin with piperine improved corpus callosum myelinated nerve fibers and oligodendrocytes (Alam et al., 2024).

Several of the quercetin nanoformulations brought about improvements in learning and memory in behavioral tests in animal models of AD (Moreno et al., 2017; Liu et al., 2020c; Amanzadeh Jajin et al., 2021). Also, they were effective in reducing oxidative stress as shown by improving malondialdehyde, glutathione, antioxidant activity to control levels in the brain (Alaqeel et al., 2022; Mobasheri et al., 2023). The levels of inflammatory markers such as TNF-α, IL-1β, and glial fibrillary acidic protein in the brain were decreased (Moreno et al., 2017; Alaqeel et al., 2022), as well as Aβ plaques and neurofibrillary tangles in the brain (Liu et al., 2020c; Rifaai et al., 2020). There was a decrease in apoptosis (Amanzadeh Jajin et al., 2021) and the number of degenerated neurons in the brain was reduced (Liu et al., 2020c; Rifaai et al., 2020).

It would be helpful to know (a) whether different nanoformulations have the same or different outcomes in a single animal model of neurodegenerative disease; (b) whether a single nanoformulation has the same or different outcomes in different animal models of neurodegenerative disease; and (c) whether treatment with nanoformulations for different times causes variations in outcomes. The main limitations to assessing these features are the number of studies reviewed and that they had for the main part used different measures of outcomes. However, choosing the AD model for which there is the largest number of reviewed studies, critical analysis indicates the following. For item (a) and from the studies using curcumin nanoformulations, five (three in mice, two in rats) had utilized Aβ peptide to induce AD (**Table 1**). One of the studies (Gutierrez et al., 2021) used lipid core nanocapsules loaded with curcumin, meloxicam, or meloxicam and curcumin combination in male mice 3 months of age. Regarding the effect on behavioral deficits, no improvement in memory was found by lipid core nanocapsules loaded with curcumin. However, curcumin liquid core nanocapsules given to male mice 3 months of age reversed a depressant-like behavior (Fidelis et al., 2019), and curcumin liquid core nanocapsules given to mice 18–22 months of age decreased the cognitive deficits in the Morris water maze test (Giacomeli et al., 2019). One of the two studies in male rats at 6–8 weeks of age found that the cognitive deficits in the Morris water maze test were decreased by curcumin-loaded nanostructured lipid carriers (Sadegh Malvajerd et al., 2019). In the other study in rats 4 weeks of age, the Aβ peptide-induced cognitive deficits were reversed by curcumin-PLGA-nanoparticles (Tiwari et al., 2014). Regarding the effect on neural deficits in the mouse studies, and allowing for different outcomes that were measured, no reduction in Aβ increased COX-2 protein expression level in the prefrontal cortex occurred with lipid core nanocapsules loaded with curcumin (Gutierrez et al., 2021). By contrast, curcumin liquid core nanocapsules reversed Aβ induced changes in superoxide dismutase (SOD) and CAT activities in the prefrontal cortex (Fidelis et al., 2019) and reversed the Aβ induced increases in inflammatory cytokines in the prefrontal cortex and hippocampus (Giacomeli et al., 2019). In the rat studies, curcumin-loaded nanostructured lipid carriers decreased the Aβ induced levels of malondialdehyde and reactive oxygen species and reduced the number of Aβ plaques in the hippocampus CA1 region (Sadegh Malvajerd et al., 2019). Curcumin-PLGA-nanoparticles inhibited Aβ-induced neurodegeneration in the hippocampus (Tiwari et al., 2014). These findings point to curcumin lipid core nanocapsules being much less effective in reducing Aβ induced behavior and neural deficits than curcumin liquid core nanocapsules, curcumin-loaded nanostructured lipid carriers, or curcumin-PLGA-nanoparticles. Interestingly, curcumin nanoparticles given to Tg2576 transgenic mice at 9 months of age resulted in a decrease of Aβ plaques in the hippocampus (Cheng et al., 2013). Increased accumulation of neurofibrillary tangles in the cerebral cortex and hippocampus of rats at 8–9 weeks of age treated with streptozotocin was decreased by curcumin nanoparticles (Noor et al., 2022). These observations suggest that curcumin liquid core nanocapsules and curcumin nanoparticles are effective in mouse and rat models of AD.

Regarding item (b), curcumin nanoparticles were tested in AD models and caused a decrease in behavioral and neural deficits (Cheng et al., 2013; Noor et al., 2022; described above). Solid lipid curcumin nanoparticles in HD models caused improvements in learning and memory (Gharaibeh et al., 2020) and increased glutathione and SOD, and decreased reactive oxygen species, levels in the brain (Sandhir et al., 2014). Curcumin SNEDDS improved learning and memory in a model of AD (Ahmad and Hafeez, 2023) and increased hippocampal SOD, glutathione, and glutathione peroxidase, and lowered TNF-α and cyclooxygenase-2 levels in a model of multiple sclerosis (Alam et al., 2024). Thus, it seems that individual curcumin nanoformulations brought about positive changes in different animal models of neurodegenerative disease.

Regarding item (c), most of the studies had treated animals with curcumin nanoformulations for 2 to 3 weeks; one study had treated for 4 days (Sadegh Malvajerd et al., 2019) and one for 7 days (Wang et al, 2019). There is an insufficient number of studies to assess whether short-term or longer-term treatment leads to a variation in outcome measures.

For quercetin nanoformulations, regarding item (a), three of the studies used aluminium chloride to induce AD in rats (**Table 2**). In terms of effect on behavioral deficits, quercetin-conjugated superparamagnetic iron oxide nanoparticles improved the memory of male rats at 7 weeks of age in the Morris water maze test (Amanzadeh Jajin et al., 2021). Regarding the effect on neural deficits, quercetin nanoemulsion given to male rats at 5–6 weeks of age reversed the effect of aluminium chloride on the levels of SOD and glutathione in the brain and decreased inflammatory cytokines (Alaqeel et al., 2022). Quercetin nanoparticles given to male rats at 6–8 weeks of age reversed the detrimental effect of aluminium chloride on hippocampal neurons and when given as a prophylactic reduced the development of Aβ plaques associated with aluminium chloride administration (Rifaai et al., 2020). Interestingly, quercetin-loaded nanoparticles improved the memory of SAMP mice 5 months of age in the Morris water maze test and reduced two different markers of brain inflammation (Moreno et al., 2017). It would seem that quercetin nanoparticles are effective in rat and mouse models of AD. Regarding items (b) and (c), there is an insufficient number of studies to perform an analysis.

Several important limitations have been identified in the studies reviewed: (1) in many of the studies there were very small sized groups of animals, often with only 3–5 animals per group; (2) no power calculations were performed to determine what group size is needed to avoid type I and type II errors; (3) where gender was reported, the groups were mainly composed of males; this is significant as, in humans, females are more at risk of developing AD; (4) where age was reported, the groups were mainly composed of young animals and only five studies had used aged animals; neurodegenerative diseases have an increased prevalence in elderly human populations; (5) there were no studies of treatment of animal models of PD and HD using nanoforms of quercetin.

In summary, the studies reviewed have shown that nanoforms of curcumin were effective in improving behavior and reducing the learning and memory deficits of animal models of AD, PD, and HD. In addition, they had a neurorestorative effect on these animal models. Nanoforms of quercetin also improved the behavior, learning and memory of animal models of AD and had a neurorestorative effect. Future studies are warranted with much larger-sized animal groups to avoid introducing statistical errors, as studies with low sample size and ultimately low statistical power are likely to introduce a type I or type II error (Schreffler and Huecker, 2024). It would be advantageous to use recommended standardized outcome measures in the animal models, so that the findings from future studies could more easily be compared. This may include behavioral tests, biochemical and histoimmunochemical assays of brain tissues, and blood/brain tissue biomarkers. MicroRNA biomarkers have recently been reported for animal models of AD (Walgrave et al., 2021), PD (Shaheen et al., 2024), HD (Martinez and Peplow, 2021), multiple sclerosis (Martinez and Peplow, 2020), and curcumin has been reported to regulate microRNA expression (Abdul-Rahman et al., 2024; Chahardehi et al, 2025). Interestingly, the upregulated level of microRNA-146a in the brain tissue of transgenic mouse models of AD was inhibited by curcumin (Li et al., 2011). Also, in a clinical case study involving 20 human patients with AD, treatment with curcumin resulted in a decrease in the blood plasma level of microRNA-146a (Gong and Sun, 2020). Curcumin was shown to modulate microRNAs involved in the regulation of the AD-associated apolipoprotein E gene (Momtazi et al., 2016) and regulate the increased levels of microRNA-128 and microRNA-9 (Patil et al., 2013).

Clinical trials should be performed with nanocurcumin and nanoquercetin, and several nanoformulations of curcumin and quercetin are available. For instance, curcumin nanoparticles were purchased from One Planet Nutrition Company, Florida, USA (Noor et al., 2022) and liposomal-formulated curcumin (Lipocurc) was obtained from SignPath Pharma Inc., Pennsylvania, USA (Chiu et al., 2013). In addition, a liposomal curcumin with resveratrol is available from UltimateVitality, Hwy Boynton Beach, Florida, USA. Recent advances in manufacturing quercetin nanofornulations have been described (Tomou et al., 2023). A search of the ClinicaTrials.gov website showed that eight studies were registered for treating AD with curcumin. Of these, NCT01001637 used solid-lipid curcumin particles (SLCP or Longvida), NCT01811381 a novel formulation of curcumin, which is better absorbed and more efficiently transported into the brain, and NCT04149639 a neuroQ supplement. No clinical trials with quercetin were indicated as having been performed. Future clinical trials could be performed using the currently available nanoforms of curcumin and quercetin, possibly in combination. Additional studies with larger-sized groups of animals, as well as the inclusion of aged animals and female subjects to better model human populations, would aid the translation of this important research to clinical practice.

## Data Availability

*Not applicable*.
